# Relationship between communication manners of head nurses with job satisfaction of nurses under their supervision in educational hospitals of Isfahan University of Medical Sciences in 2006

**Published:** 2010

**Authors:** Abdollah Rezaei Dehaghani, Habibollah Hosseini, Khosrow Tavakol, Soheila Bakhtiyari

**Affiliations:** * Department of Health Nursing, School of Nursing and Midwifery, Isfahan University of Medical Sciences, Isfahan, Iran; ** Department of Operating Room Nursing, School of Nursing and Midwifery, Isfahan University of Medical Sciences, Isfahan, Iran

**Keywords:** Nurses, communication, job satisfaction

## Abstract

**BACKGROUND:**

Interpersonal communication is considered as an important and effective factor of job satisfaction and efficiency and has special significance in nursing career because of the face to face relationship with patients. This study aimed to determine the association between head nurses’ interpersonal communication and job satisfaction of nurses under their supervision. The study was conducted in educational hospitals of Isfahan University of Medical Sciences in 2006.

**METHODS:**

This was a descriptive and analytical study on 203 nursing personnel working in educational hospitals of Isfahan University of Medical Sciences in 2006. Data were collected using Job Descriptive Index (JDI) developed by Smith and Kendall and interpersonal communication was measured using a researcher-made questionnaire. Data were analyzed using SPSS software and Pearson’s test and presented in tables and diagrams.

**RESULTS:**

The majority of the participants (148 subjects, 73%) believed that head nurses’ interpersonal communication was excellent and in general Pearson’s test showed a significant association between head nurses’ interpersonal communication and their personnel’s job satisfaction (p < 0.011).

**CONCLUSIONS:**

Based on the results of this study on the relationship between interpersonal communication of the head nurses and job satisfaction of their personnel, we can improve the job satisfaction of nursing personnel as well as patients’ satisfactory and level of services by developing educational courses and workshops on importance and effectiveness of interpersonal communication for head nurses.

Monjamed et al. (2004) in their study believed that job satisfaction shows people’s positive or negative attitude towards their job and it is affected by several factors. Quoting from Chang et al, they described job satisfaction as the collection of people’s emotions towards their job. They said that from the viewpoint of Herzberg, factors leading to job satisfaction are differentiated from those lead to job unsatisfactory including health factors or external factors in one group and motivation and internal factors in the second group.^1^

Also Khoda Rahimi et al (2006) believed that job satisfaction has a complex meaning and have psychological, physical and social dimensions. According to them, it is not just one factor that brings job satisfaction, but a special combination of various factors makes people feel job satisfaction at a specific time of their life. The emphasis a person puts on factors such as income, job’s social status, work environment conditions and job productions at a certain time of life leads to the job satisfaction (quoted from Shafiabadi, 2000).^2^

People including health care personnel or staff of any other organization have some needs and wishes, which they try to achieve. One of the ways to achieve these wishes is selecting a job and career. It seems that most people who select jobs such as medical practice, nursing, midwifery, social working, paramedical etc enjoy serving human being. These people achieve job satisfaction when they can directly serve patients and answer to their needs. But the manager of nursing services should not be simple minded and imagine that nursing unit personnel including nurses, midwives and paramedics do not care for the financial or status related aspects of their career. But by considering the needs and human characteristics, they should divide workloads in a way that make their personnel satisfied with their jobs. There is no doubt that managers can never make all their personnel happy with everything. This is even more difficult in nursing and midwifery careers due to limited organizational positions and possibility of promotions.^3^

Nurses’ job satisfaction is not just their satisfaction with the income, but the joy they get from doing their job is involved in it and it is related to good relationship with colleagues, existence of suitable conditions for serving and the joy of doing specific kind of nursing services.^4^ Job satisfaction effects efficiency, quality of service and personnel’s retention. Monjamed et al. (2004) quoted from Chang that job satisfaction is an important factor for performance and quality of services provided by hospital staff including nurses. They continued that Tylor (1975) in his study in the US showed that nurses’ job satisfaction is associated with improving quality of patient care and increasing their productivity. Job satisfaction itself determines many organizational variables such as increasing productivity as well as the work quality and quantity.^1^

Interpersonal relationships are probably more important than any other factors in job satisfactory. People want to work with those they like and can get along with.^5^ Interpersonal relationships are about relationship or positive and purposive relationships between two or more human beings. Interpersonal relationship is the essence of human existence and values and it is the social behavior phenomena of human groups whose shape and kind differentiate human from animals. Interpersonal relationship in this meaning is the constructive and encouraging factor. Each person has to create various relationships in life environment. Each person in family, school, work place, entertainment, praying, etc. needs relationship with others.^6^

Jafari (2005) defined interpersonal relationships as human beings’ ability to relate with others by accepting their existence, personality and characteristics as well as their possible differences with the manager’s personality.^6^ Kazmier (1991) believed that interpersonal relationship is a new discipline in scientific studies to improve efficiency and work spirit and it is not considered a problem any more.^7^

Creating relationships in organizations is a difficult process as both organizational and interpersonal dimensions should be considered. Understanding the common obstacles in relationship dimensions in health care institutions and finding the steps toward removing those obstacles can improve new skills to create effective relationship.^8^

In this regards, O’shea (2007) described some skills to create effective relationship as follow: create the suitable atmosphere for constructive relationship; help staff to understand the significant issues; slowly specify performance problems for them and describe the duty; ask them about their problems and listen to them in a friendly manner; involve them in problem solving process and ask them to suggest solutions; avoid pointless advices; and try to reach a mutual agreement about activities to be performed and followed up.^9^

Regarding human relationships, interpersonal relationships are mentioned. It may be said that creating good human relationship is one of the most important factors for achieving goals. On this basis, to achieve one’s goals, every human needs to be able to relate to others and this ability is called managing human relationships in management.^10^

Considering above, which express the importance of job satisfaction and its role in increasing efficiency especially in nursing career as well as the significant role of human relationship in creating and improving satisfaction, and also based on the researcher’s several years of experience, this study was conducted to determine the association between interpersonal relationships of head nurses with the job satisfaction of the personnel working under their supervision in educational hospitals of Isfahan University of Medical Sciences in 2006. The objectives of this study include: 1) determining personal data of nurses working in educational hospitals, 2) determining the interpersonal relationship manner of the head nurses from the viewpoint of nursing personnel, 3) determining the job satisfaction of nursing personnel, and 4) determining association between head nurses interpersonal relationship manner from the viewpoint of nursing personnel with the job satisfaction of nurses.

## Methods

This was a descriptive analytical study conducted in 2006. The study population included all nursing personnel of educational hospitals of the Isfahan University of Medical Sciences including nurses with college degree, undergraduate and graduate degrees who had at least 6 months of work experiment in the wards of above mentioned hospitals as nursing personnel. Variables included interpersonal relationship, age, sex, work experiment, education and job satisfaction. Study environment included hospitals of the Isfahan University of Medical Sciences in 2006. The sample size was calculated considering the standards and included 203 nurses with college degree, Bachelor and Master degrees, who were working in hospitals of Isfahan University of Medical Sciences and had the entry criteria. The process of the study was as follow: the hospitals and sample size were identified and the researcher obtained letter of introduction and referred to the study places during a time of personnel’s less workload. After explaining study objectives to the samples, they completed the questionnaire. Data were entered a computer using SPSS software and were analyzed using descriptive and inferential statistical tests (Pearson). The results were presented in tables and diagrams. Data collection instrument was a questionnaire which included 3 sections. The first section was demographic data (sex, age, education, work experiment, etc.); the second section was a researcher made questionnaire for interpersonal relationship manners of the head nurses; and the third section was the standard questionnaire of job satisfaction developed by Smith and Kendall. The validity of the interpersonal relationship questionnaire (second section) was proved by content validity. First, a draft was prepared based on books and articles published on human relationships and the quality of content was checked and evaluated by professionals and the faculty members of the Isfahan Nursing and Midwifery College and College of Educational Studies. Then, based on the suggestions, the questionnaire was edited and refined. To determine the reliability of the questionnaire, retest was used. The third section of the questionnaire was the standard test of Smith and Kendall, whose validity and reliability was already approved. To analyze data, descriptive and inferential methods (correlation coefficient and Pearson) were used. The researcher tried his best to follow ethical codes, some of which will be mentioned here. The researcher obtained a letter of introduction from the affiliated college and introduced himself to all the related personnel in the centers. The units were free to decide if they wanted to participate in the study or not. To get the trust of units, their names were not requested in the questionnaire. The results of the study will be provided to the authorities of the centers if they want.

## Results

The results showed that a high percentage of participants (77%) were female nurses (23% male nurses). Considering the job status, 90.5% had a BS degree. Also, the longest working experiment was 1 to 5 years (31.5%) for nursing personnel and 11-15 years (50.3%) for the head nurses. It means that personnel and head nurses’ job experiment, sex and educational degree had a good distribution. For the second objective of the study that was “determining the interpersonal relationship manners of the head nurses from the viewpoint of nursing personnel in hospitals of the Isfahan University of Medical Sciences”, the results showed that 73% of the study population believed that head nurses’ interpersonal relationship manners were excellent and just 3.4% claimed it to be weak. For the third objective that was “determining the job satisfaction of nursing personnel in educational hospitals of the Isfahan University of Medical Sciences”, the results showed that majority of participants (65%) had excellent job satisfaction considering their current managers and just 2.4% mentioned it is weak. For the fourth objective that was “determining the association between head nurses’ interpersonal relationship manners from the viewpoint of nursing personnel with their job satisfaction”, the Pearson statistical test showed that there is a significant association between the two (p < 0.011).

## Discussion

The study conclusion is that in general the interpersonal relationship manners of head nurses have been appropriate. Ghabeljoo (1990) wrote: it is the head nurse who create a friendly relationship between the members of the hospital team to perform their jobs in a calm atmosphere, because not every reaction of the hospital team can benefit patients. This behaving style leads to mutual friendly behavior of the personnel.^10^

Also, the job satisfaction of the nursing personnel in relation with the head nurses and their current colleagues was good. In a study by Yaghuti aimed to assess nurses’ job satisfaction and the effective factors to provide strategies in Imam Khomeini Hospital of the Tehran University of Medical Sciences in 2004, the most had a high satisfaction of the relationship with colleagues (78.1%).^11^ From all, 64% of nursing personnel who participated in this study considered their job satisfaction good, while just 22% considered it excellent. Just 13.8% of nursing personnel said that their job satisfaction related to opportunities with organizational promotion was excellent, while 82% said that it was average or good. Considering the job satisfaction of nursing personnel with the payments (salary and bonuses) of their current job, a high percentage (47.3%) considered it weak and in total 77% considered it weak and average. In a study by Mardali in 2004 aimed to assess nurses’ job satisfaction and the effective factors to provide strategies in Imam Khomeini Hospital of the Tehran University of Medical Sciences, a total of 63% of nurses were unhappy with their job and 35.6% had average satisfaction and 1.4% were highly satisfied with their jobs.^12^ Also, in a study by Monjamed et al. in 2004, the job satisfaction of nurses in terms of their salary and bonuses was low (77.3%).^1^

In general, there was a significant association between head nurses’ interpersonal relationship manner and the job satisfaction of nurses working in Imam Khomeini Hospital of the Tehran University of Medical Sciences. Mardali’s study showed that in general the mean score of job satisfaction was low and the lowest job satisfaction was related to distribution of specific work payments and the highest satisfaction was from hospital management.^12^ Moreover, in a study by Hamidi et al. in 2006 on the stress level and job satisfaction of Hamadan health care centers’ personnel, the results showed that majority of health care personnel were satisfied by their managers or bosses.^13^ In this regards, Monjamed et al. found that the commonest cause of nurses’ resigning and job unsatisfactory (70.6%) was related to authorities’ lack of attention to their suggestions and opinions as well as lack of support from their managers and head nurses.^1^

## Conclusions

The study conclusion is that first, in general the interpersonal relationship manners of head nurses have been appropriate. Second, the job satisfaction of the nursing personnel in relation with the head nurses and their current colleagues was good. And third there was a significant association between head nurses’ interpersonal relationship manner and the job satisfaction of nurses working under their supervision.

Considering the results showing the association between head nurses’ interpersonal relationship manner and the job satisfaction of nurses working under their supervision, it is recommended that by planning continuing educational programs, holding workshops and lectures, providing pamphlets, CDs and booklets about the importance and effectiveness of interpersonal relationships and improving head nurses knowledge of that, the job satisfaction of nursing personnel can be increased, which in turn leads to patients’ satisfaction and improvement of health care service quality.

The authors declare no conflict of interest in this study. Ethical committee approved the study.
